# Attitudes towards mental illness in Malawi: a cross-sectional survey

**DOI:** 10.1186/1471-2458-12-541

**Published:** 2012-07-23

**Authors:** Jim Crabb, Robert C Stewart, Demoubly Kokota, Neil Masson, Sylvester Chabunya, Rajeev Krishnadas

**Affiliations:** 1 Forth Valley Royal Hospital, Stirling Road, Larbert, UK; 2 Department of Mental Health, College of Medicine, University of Malawi, Blantyre, Malawi; 3 Scotland-Malawi Mental Health Education Project, c/o Royal Edinburgh Hospital, Edinburgh, UK; 4 Wishaw General Hospital, Netherton St, Wishaw, UK; 5 Sackler Institute of Psychobiological Research, Section of Psychological Medicine, Southern General Hospital, Glasgow, UK

## Abstract

**Background:**

Stigma and discrimination associated with mental illness are strongly linked to suffering, disability and poverty. In order to protect the rights of those with mental disorders and to sensitively develop services, it is vital to gain a more accurate understanding of the frequency and nature of stigma against people with mental illness. Little research about this issue has been conducted in Sub- Saharan Africa. Our study aimed to describe levels of stigma in Malawi.

**Methods:**

A cross-sectional survey of patients and carers attending mental health and non-mental health related clinics in a general hospital in Blantyre, Malawi. Participants were interviewed using an adapted version of the questionnaire developed for the “World Psychiatric Association Program to Reduce Stigma and Discrimination Because of Schizophrenia”.

**Results:**

210 participants participated in our study. Most attributed mental disorder to alcohol and illicit drug abuse (95.7%). This was closely followed by brain disease (92.8%), spirit possession (82.8%) and psychological trauma (76.1%). There were some associations found between demographic variables and single question responses, however no consistent trends were observed in stigmatising beliefs. These results should be interpreted with caution and in the context of existing research. Contrary to the international literature, having direct personal experience of mental illness seemed to have no positive effect on stigmatising beliefs in our sample.

**Conclusions:**

Our study contributes to an emerging picture that individuals in Sub-Saharan Africa most commonly attribute mental illness to alcohol/ illicit drug use and spirit possession. Our work adds weight to the argument that stigma towards mental illness is an important global health and human rights issue.

## Background

Stigma can be defined as a sign of disgrace or discredit, which sets a person apart from others [1]. The experience of stigma is characterized by shame, blame, secrecy, being the “black sheep of the family”, isolation, social exclusion and discrimination. The stigma and discrimination associated with mental illness has been strongly associated by the World Health Organisation (WHO) with suffering, disability and poverty [2]. It is a major barrier to treatment and the prevention of suicide [1,3]. Stigma is also a major reason why sufferers of mental illness fail to acknowledge their illness and it has been described as the underlying factor mitigating against the social re-integration of those recovering from mental illness [4,5].

Early work in the field suggested that stigma might be less common in Africa, particularly in Muslim countries [6]. Evidence to discredit this view (or “contrary to this view”) began to emerge from Islamic states such as Morocco where stigma was found to be a major burden to families [7]. In general there is a paucity of research available concerning attitudes towards mental illness in Africa and what there is tends to be drawn from vastly different areas of the continent. It has been suggested that the early observations about a lack of stigma in Africa were due to a lack of research on the ground rather than a more culturally receptive attitude to mental illness [2]. Indeed more recent studies from across Africa have suggested that the experience of stigma may be prominent in individuals with mental illness, their family and the community at large. Studies conducted in Ethiopia, in Eastern Africa suggest that the experience of stigma by people with mental illness may be widespread with three quarters of family members of individuals with a mental illness experiencing stigma [8]. In surveys of community attitudes to mental illness in South Africa, members of the general public have been found to attribute mental illness to stress or a lack of willpower rather than a medical illness [9].

Most work on community attitudes has been done in West Africa and has demonstrated poor knowledge regarding causation, widespread negative views towards mental illness and an overwhelming majority believe that those with mental illness are dangerous and unsuitable for normal social contact [10]. In those attending outpatient clinics in Nigeria ‘supernatural’ reasons were found to be the most popular explanations for mental illness amongst both carers and patients whilst ‘psychosocial’ explanations were least popular, a notable difference from international findings [11,12]. Family members in Nigeria have also been found to experience a higher frequency of anger and stigma [13]. It is difficult to generalise attitudes towards mental illness even within the same geographical region of the African continent. For example, in nearby Ghana there seems to be a greater reliance in rural areas on culturally specific explanations allied with more acceptance and support [14].

In order to best protect the rights of those with mental disorders and to sensitively develop services, it is vital to gain a more accurate impression of the frequency and nature of stigma across Sub-Saharan Africa. Our study aimed to explore the frequency and characteristics of stigmatising beliefs towards mental illness in Malawi, a country in South East Africa.

The most up to date figures from the World Health Organisation suggest mental disorders are the forth most common cause of disability in Malawi after HIV/AIDS, cataracts and malaria [15]. To meet this need the country has a mental health provision of 2.5 psychiatric nurses and less than 1 psychiatrist per 100,000 population [16]. The overwhelming majority of mental health treatment is provided in primary care. Practitioners working in primary care in Malawi are paramedics who are known as medical assistants and clinical officers. Medical Assistants undergo two years of medical training i.e. one year of theory and one year of clinical attachments and graduate with a certificate in medicine. They are the backbone of primary care in Malawi. As part of their undergraduate training medical assistants undergo two weeks of theory teaching in psychiatry and two weeks of clinical attachment at the main psychiatric hospital [17].

## Methods

The study was conducted in Queen Elizabeth Central Hospital, a tertiary teaching hospital in Blantyre, the second most populous city in Malawi.

A cross-sectional survey was conducted over a 2-week period. A single member of the research team recruited participants (DK). All participants were given an information sheet and written informed consent was obtained before entry to the study. Participants self-completed the questionnaire. Data was collected from consecutive attendees (patients and their carers) in the waiting room of epilepsy, psychiatry, general medical and surgical outpatient clinics. Carers and patients were surveyed individually (carers did not complete the questionnaire on behalf of patients). Those individuals unable to read or write, or who were too unwell to complete the questionnaire, were excluded. The study was approved by the College of Medicine Research Ethics Committee (COMREC), University of Malawi.

Participants were interviewed using the questionnaire developed for the World Psychiatric Association Program to Reduce Stigma and Discrimination Because of Schizophrenia [18]. This tool was developed to measure stigma internationally. It consists of 17 dichotomous questions regarding the causes of mental illness, views about mental illness and social distance practices related to mental illness. As with other published studies we adapted the questionnaire for use across the spectrum of mental illness by replacing the term ‘schizophrenia’, with ‘mental illness’ [10]. The questionnaire was translated into local dialects and approved by a local panel of mental health professionals and service users. Although interviewees were anonymous, their demographic details were recorded.

Data was analysed using SPSS. The questionnaire used in our study does not produce a total score for stigma therefore analysis focused on the outcome of individual questions as has been the case in other published work [10]. Chi-square tests were performed on categorical data and independent student’s t-tests on continuous outcomes.

## Results

A total of 210 participants completed our survey. Three people refused, 15 did not have the capacity to complete the questionnaire due to the severity of their illness and 15 were non-literate. The mean age of the sample was 33.9 years (SD 13.5). The gender distribution was 42.4% male and 57.6% female. See Tables 1 &2 for further clinical and demographic details.

**Table 1:** Description of those attending clinic and type of clinic attended

		**Percentage**	**Number**
Type of clinic attendee	Patient	62.9	132
	Guardian	37.1	78
Type of clinic attended	Psychiatry and Epilepsy clinic	47.1	99
	Other clinic	52.9	111

**Table 2:** Demographic details of participants

**Demographic**		**%**	**N (210)**
Sex	Male	42.4	89
	Female	57.6	121
Educational status	Educated to primary school level	41	86
	Educated beyond primary school level	59	124
Poverty	Yes (earning less than $1 per day)	43.3	91
	No (earning more than $1 per day)	56.7	119

Most participants attributed mental disorder to alcohol and illicit drug abuse (95%). This was closely followed by brain disease (92.8%), spirit possession (82.8%) and then by psychological trauma (76.1%). Other factors, whilst potentially important were attributed as causes of mental illness by less than half of our sample. Results regarding the cause of mental illness, views about mental health and social distances practices in our sample are summarised in Tables 3, 4 &5.

**Table 3:** Perceived causes of mental illness

**Causes of mental illness**	**Percentage (number) holding belief**	**Percentage (number) not holding belief**
Drug or Alcohol misuse causes mental illness	95.7 (201)	4.3 (9)
Possession by evil spirits causes mental illness	82.8 (174)	17.1 (36)
Traumatic events or shock cause mental illness	76.1 (160)	23.8 (50)
Genetic/familial causes mental illness	47.6 (100)	52.4 (110)
God’s punishment causes mental illness	21.9 (46)	77.6 (163)
Brain Disease causes mental illness	92.8 (195)	7.1 (15)
Biological factors other than brain disease or genetic inheritance cause mental illness	32.8 (69)	67.1 (141)
Poverty causes mental illness	43.3	56.7

**Table 4:** Views about mental health

**Views about mental health**	**Percentage (number) holding belief**	**Percentage (number) not holding belief**
Mentally ill patients can be treated outside the hospital	24.2 (51)	75.7 (159)
All mentally ill patients are a nuisance to the public	20.4 (43)	79.5 (167)
People with mental illness can work in a regular job	39 (82)	61 (128)

**Table 5:** Social distance practices and mental illness

**Social distance practices**	**Percentage (number) holding belief**	**Percentage (number) not holding belief**
Are you afraid to have a conversation with the mentally ill	63.3 (133)	36.7 (77)
Would you be upset or disturbed about working with the mentally ill	33.8 (71)	66.2 (139)
Would you be able to maintain a friendship with the mentally ill	68.5 (144)	31.4 (66)
Would you be unwilling to share a room with the mentally ill	40.6 (85)	59 (124)
Would you be ashamed if you were related to a mentally ill person and people knew	8.1 (17)	91.9 (193)
Would you be prepared to marry a mentally ill person	18.6 (39)	81.4 (171)

There were no significant differences in stigmatising beliefs according to gender, education and whether the subject was a patient or carer. Those participants defined as experiencing poverty were more likely to attribute mental illness to trauma or shock than their more affluent counterparts (84/91 versus 76/119 p < 0.001). Those attending non-mental health clinics were less willing to marry someone who had experienced mental illness compared to those at the psychiatric and epilepsy clinics (12/111 versus 27/99 p = 0.02). There were no other significant differences in responses between those attending psychiatry/ epilepsy and non mental health related clinics. Younger patients were significantly more likely to attribute mental illness to illicit drugs and alcohol (mean age 28.4 years versus 35.7, p = 0.001) and as God’s punishment (30.5 years versus 35, p = 0.04). Older participants were more likely to consider those with mental illness a public nuisance (37.8 years versus 32.9, p = 0.03).

## Discussion

This was the first study of attitudes toward mental illness to be conducted in Malawi. An over whelming majority of participants recruited from mental health and non-mental health clinics at a large general hospital attributed mental illness to substance misuse and spiritual causes such as spirit possession and God’s punishment [10,11,19]. Our findings are broadly consistent with those from a large and robust community survey using the same rating scale conducted in Nigeria. That work found illicit drugs and alcohol (80.8%), spirit possession (30.2%), psychological trauma (29.9%) and genetic explanations (26.5%) to be the most common attributions for mental illness [10]. That such similar results should be found is perhaps surprising considering the different study designs and samples. The Nigerian study involved multistage cluster sampling of households across three separate states whilst our work focused on consecutive attendees at hospital clinics. Though more work is required across Sub-Saharan Africa, these findings would seem to suggest that views on causation of mental illness maybe common across the region.

The fact that most of our sample attributed mental illness most strongly to drugs and alcohol might be considered a positive finding on one level, as it suggests potential treatability. However only a few mental disorders are known to be aetiologically attributable to alcohol or illicit drug use, therefore the majority view found in our sample is not factually correct. In most Sub-Saharan African societies alcohol and illicit drug use are viewed negatively and are considered due to moral failings on the part of the user. This may also be why many participants attributed mental illness to spiritual processes.

More of our sample attributed mental illness to Gods punishment compared to participants in West Africa (21.9% in Malawi compared to 9.3% in Nigeria). In Nigeria, religious-magical views of causation have been found to be more associated with negative and stigmatizing attitudes to the mentally ill compared with biological explanations [20]. Spiritual explanations have also been found for mental states due to physical illness such as delirium in this region [21]. These beliefs may also explain why many cases of mental illness in Sub-Saharan Africa are treated punitively or outside of the Western Health care systems, for example, via traditional or faith healers. Our findings regarding drugs and alcohol and spiritual matters as the most popular causes of mental illness are therefore a concern, as they reflect a potential for discrimination and non-medical treatment or at its worst, maltreatment.

One notable difference in our results from other work in West Africa is the more frequent attribution of mental illness to brain disease (92.8% in Malawi versus 9.2% in Nigeria). Once again the differences observed between Nigeria and Malawi may reflect the different populations sampled. Whilst we are not aware of any direct health promotion strategies regarding mental health and mental illness in the Malawian clinics, it seems reasonable that a population attending these services will be more attuned to a medical model of causation. The strong attribution of mental illness to brain disorders does however appear contradictory considered alongside the equally strong spiritual attributions given by participants for mental illness (such as spirit possession and mental illness being a punishment from god). While in Western traditions, the mind and body are traditionally considered as distinct entities, this may not be true in Malawi. It is possible that spiritual possession is believed to influence the brain directly. Further qualitative research is needed to better understand the culturally specific inter relationships between these explanations for mental illness.

Malawi is currently ranked 153 of 169 on the latest UN Development Index and Category E (very high mortality) on the WHO mortality register. It is therefore perhaps surprising that only around half of our sample (43.3%) endorsed poverty as a cause of mental illness. Furthermore those defined as existing in poverty did not more readily attribute mental illness to poverty than their more affluent counterparts. The reasons for this may be two fold. If most of a population exists in relative poverty, then it may be hard for those within the society to consider poverty as a contributing factor for an illness which only affects a minority. In developed economies, though a diagnosis of mental illness can carry stigma, it can also entail sympathy (from some quarters), treatment from established health services and support from the welfare state. These factors are far from the established norm in Malawi where destitution may await those with mental illness. It is possible that those in a state of poverty are reluctant to consider that they may be at risk of an even worse fate.

Only a quarter of our respondents believed mental illness could be treated outside of the hospital setting. On one level this is to be expected as around half our sample consisted of patients and their carers attending established mental health clinics at a major teaching hospital in an urban centre. The population in our sample may therefore have been self selected to have a more positive or biased view towards hospital treatment. However our finding may reflect the reality that there is very little community mental health care in Malawi. Respondents may simply not have been aware of any alternative to treatment in a hospital. They may also have had concerns about the reality of treatment in a less specialised centre.

With regards to social distance and mental illness, very few of our respondents would have been ashamed if someone in their family experienced mental illness and most were prepared to maintain a friendship with someone who had been mentally ill. However, our respondents seemed to be less prepared to consent to increasing social intimacy with someone who had experienced mental illness. Less than half were prepared to share a room with someone who had experienced mental illness and only approximately one in five was prepared to consider marriage. Since genetic factors were believed by half of our participants to be a cause of mental illness, fears about mental illness being passed on to future offspring may have influenced these findings.

Our study found less stigmatising beliefs in terms of social distance compared with Nigerian samples (8.1% of our sample compared with 82.9% in Nigeria). This may be explained by the fact that half of our sample had either personally experienced mental illness or were related to someone who had. Promoting direct personal contact between individuals experiencing mental illness and the general public has been shown to reduce stigma [22-24]. Stigma / increased social distance have also been found to be correlated with a lack of personal contact with mental illness in three Nigerian studies [25-27]. It is possible that many in Gureje’s Nigerian community sample had not had this personal experience compared to our population. This does not however explain why we found no difference in stigma scores between psychiatric and non psychiatric clinic attendees. According to the available evidence it would be expected that those attending psychiatric clinics would have had more personal experience of living with mental illness and so express less stigmatising beliefs than those participants attending medical and surgical clinics. In Nigeria psychiatric patients have been found to experience high self stigma rates of up to 21.6% and this phenomenon may explain the lack of difference in stigma scores found between mental health and non mental health clinic attendees in our results [28].

Our study has some limitations. It is possible that our sample population, consisting of literate persons attending an urban centre teaching hospital may not be representative of Malawi as a society and so limits the generalisability of our results. The role of demographic variables in stigma (and therefore any potential bias in our sample) is far from clear from the existing research that has been performed in Africa. Studies from Nigeria, Ghana and Ethiopia suggest that urban dwelling and higher education correlates with biological/ psychological attributions for mental illness and lower stigma scores [25,29,30]. However, the most robust study in West Africa by Gureje et al found no correlation between any demographic variable, including urban, semi rural and rural dwelling [10]. Any associations between stigmatising beliefs and demographic groupings found in our study therefore need to be interpreted with extreme caution. Though it is possible that older participants were indeed more conservative and so were more likely to consider those with mental illness a nuisance, and that younger participants might have had more experience of alcohol and illicit drugs as a direct cause of mental illness (as was shown in our results), it needs to be borne in mind that we found no consistent differences in stigmatising beliefs between demographic groups, only single question associations. Ultimately we can only speculate as to why participants held the particular views or attitudes regarding mental illness they expressed in our study. This is due to the limitations of a quantitative study design. Whilst our study is an important first step in clarifying what patients and their carers think about mental illness in Malawi further qualitative work is required to deepen our understanding of this important issue.

## Conclusions

There is a marked discrepancy in explanatory models of mental illness between Africa and the other parts of the world. In a review of the literature the general public internationally were found to prefer psychosocial to biogenetic explanations for mental illness [12]. Our study contributes to an emerging picture that this is not the case in Africa where most individuals attribute mental illness to alcohol/ illicit drug use and spiritual causes. Our work continues to add weight to the argument that stigma towards mental illness exists across the globe, including Africa where unique culturally appropriate interventions will need to be developed.

## Abbreviations

UN, United Nations; WHO, World Health Organisation.

## Competing interests

Authors of this manuscript have no competing interests.

## Authors’ contributions

The study protocol was devised by JC & SC. Ethical approval was submitted by SC. Data collection was completed by DK and supervised by RS. Data analysis was performed by JC and RK. The study was written up by JC, RK, RS, DK & NM. All authors have given final approval of the version to be published.

## Authors’ information

Robert Stewart (RS) is a Lecturer and Consultant Psychiatrist at the College of Medicine, University of Malawi, Blantyre. He is supported in his work helping develop mental health services in Malawi by the Scotland Malawi Mental Health Education Project (SMMHEP). The mission of this UK registered charity (Scottish Charity number SC039523) is to improve the training of mental health workers in Malawi. Demoubly Kokota (DK) is a psychology graduate of University of Malawi and is currently employed as Research Coordinator, SMMHEP project in Malawi. Jim Crabb (JC) & Neil Masson (NM), two Consultant Psychiatrists based in Scotland volunteer with SMMHEP and have visited Malawi to assist with SMMHEP’s training and research projects. Sylvester Chabunya (SC) is a medical student who received his undergraduate psychiatry teaching from SMMHEP volunteers. He displayed an interest and aptitude in mental health after this experience and expressed an interest in gaining more experience in mental health research. Rajeev Krishnadas (RK) is a Clinical Lecturer at the University of Glasgow. He has kindly volunteered his time and expertise in research to assist this paper. All authors have given final approval of the version to be published.

## Conflict of interest

No authors have any conflict of interest to declare.

## Funding

SMMHEP is a registered charity that receives funding from the Scottish Government & private donations. DK is employed by University of Malawi with salary funding from SMMHEP. JC, NM and RS’s flights to Malawi whilst they were volunteering for training and research projects were reimbursed by SMMHEP. For more information on the work of SMMHEP please visit the following link http://www.smmhep.org.uk/.

## Pre-publication history

The pre-publication history for this paper can be accessed here:

http://www.biomedcentral.com/1471-2458/12/541/prepub
